# Complications neurologiques de la dengue chez l’adulte dans les CHU de Ouagadougou au cours de l’épidémie de 2023 au Burkina Faso

**DOI:** 10.48327/mtsi.v6i1.2026.794

**Published:** 2026-01-07

**Authors:** Djingri Labodi LOMPO, Sylvie D KAGAMBEGA, OUATTARA Souleymane, ZOUNGRANA Alassane, Melody Zeinab GNAMPA, Fabienne Yabtouta KERE, NAPON Christian, MILLOGO Athanase

**Affiliations:** 1 Université Joseph Ki-Zerbo, UFR/SDS, Département de neurologie, Ouagadougou, Burkina Faso; 2 CHU Tengandogo, Service de neurologie, Ouagadougou, Burkina Faso; 3 CHU Bogodogo, Service de neurologie, Ouagadougou, Burkina Faso

**Keywords:** Épidémie, Dengue, Complications neurologiques, Encéphalopathie, Encéphalite, AVC, Guillain-Barré, Létalité, Ouagadougou, Burkina Faso, Afrique subsaharienne, Epidemic, Dengue, Neurological complications, Encephalopathy, Encephalitis, Stroke, Guillain-Barré syndrome, Lethality, Ouagadougou, Burkina Faso, Sub-Saharan Africa

## Abstract

**Introduction:**

La dernière épidémie de dengue survenue en 2023 au Burkina Faso a causé la morbimortalité la plus importante d’Afrique. Les complications neurologiques de la dengue sont décrites comme associées à une létalité importante. Nous avons réalisé le présent travail dans le but d’étudier ces complications chez les adultes hospitalisés dans les CHU de Ouagadougou durant l’épidémie de dengue de 2023, au Burkina Faso.

**Patients et méthodes:**

Il s’est agi d’une étude transversale, rétrospective, descriptive et analytique, ayant concerné les cas confirmés de dengue chez des adultes hospitalisés du 1^er^ janvier au 31 décembre 2023 dans les CHU de Ouagadougou. La fréquence des complications neurologiques de la dengue, les données sociodémographiques, cliniques, paracliniques et évolutives intrahospitalières de ces patients ont été analysées. Une analyse bivariée puis une étude multivariée selon la méthode de régression logistique multiple ont permis d’identifier les facteurs associés à la létalité intrahospitalière de ces patients.

**Résultats:**

Au total, 1 057 patients ont été hospitalisés pour une dengue dans les CHU de Ouagadougou durant la période épidémique, dont 116 (11 %) ont présenté des complications neurologiques, avec un âge moyen de 47,6 ans et une prédominance féminine (51,7 %). Les troubles de la conscience, les crises épileptiques, les troubles psycho-comportementaux, dans respectivement 84,5 %, 20,7 % et 14,6 % des cas, étaient les signes cliniques neurologiques les plus fréquemment retrouvés. Les complications neurologiques rencontrées étaient les encéphalopathies (7,8 %), les encéphalites probablement dues à la dengue (0,8 %), les accidents vasculaires cérébraux (0,7 %) et le syndrome de Guillain-Barré (0,1 %). La létalité hospitalière a été de 69 % (80 patients) et l’insuffisance rénale aiguë a été le seul facteur indépendant qui lui a été associé (OR = 3,49; p = 0,02).

**Conclusion:**

Au Burkina Faso, les complications neurologiques de la dengue sont dominées par les encéphalopa-thies multifactorielles, grevées par une très lourde mortalité. Le dépistage et l’admission précoces dans les unités de soins intensifs ou de réanimation des patients ayant une dengue sévère (avec notamment des signes neurologiques), contribueraient à améliorer leur pronostic.

## Abstract (en)

**Introduction:**

The most recent dengue epidemic in Burkina Faso in 2023 resulted in the highest morbidity and mortality rates in Africa. Neurological complications of dengue are associated with high mortality rates. This study investigated these complications in adults hospitalized at the Ouagadougou University Hospital during the 2023 dengue epidemic in Burkina Faso.

**Patients and methods:**

This cross-sectional, retrospective, descriptive, and analytical study examined confirmed cases of dengue fever in adults hospitalized from January 1 to December 31, 2023, at the University Hospitals of Ouagadougou. We analyzed the frequency of dengue fever-related neurological complications and the sociodemographic, clinical, paraclinical, and intra-hospital data of these patients. A bivariate analysis, followed by a multivariate study using multiple logistic regression, identified factors associated with in-hospital mortality.

**Results:**

A total of 1,057 patients were hospitalized with dengue fever in Ouagadougou’s university hospitals during the epidemic period. Of these patients, 116 (11%) presented with neurological complications. The mean age of these patients was 47.6 years, and 51.7% were female. The most frequently found neurological clinical signs were impaired consciousness (84.5%), epileptic seizures (20.7%), and psycho-behavioral disorders (14.6%). The neurological complications encountered were encephalopathies (7.8%), probably dengue-related encephalitis (0.8%), strokes (0.7%), and Guillain-Barré syndrome (0.1%). The in-hospital mortality rate was 69% (80 patients), and acute renal failure was the only independent factor associated with it (OR = 3.49; p = 0.02).

**Conclusion:**

In Burkina Faso, the neurological complications of dengue are primarily multifactorial encephalopathies, which are associated with a high mortality rate. Screening patients with severe dengue early and admitting them to intensive care or resuscitation units (particularly those with neurological signs) would improve their prognosis.

## Introduction

La dengue est une maladie infectieuse due à *Orthoflavivirus denguei* (Dengue virus, DENV), transmise par les moustiques *Aedes aegypti* et *Ae. albopictus* [23]. Elle est endémique dans les régions à climat tropical et subtropical et très répandue, avec une incidence annuelle en progression constante dans le monde, atteignant 100 à 400 millions d’infections par an. On estime que la population mondiale à risque atteindra 4,7 milliards de personnes supplémentaires d’ici à 2070. En Afrique subsaharienne en général et au Burkina Faso en particulier, la dengue est endémo-épidémique et en pleine expansion du fait des changements climatiques, de l’expansion géographique des vecteurs, de l’augmentation des déplacements humains, de l’urbanisation rapide et non planifiée, du manque de contrôle vectoriel durable, de la co-circulation et de l’émergence de plusieurs sérotypes (DENV 1 à 4) [10,14].

La dengue se présente généralement sous la forme d’une maladie fébrile habituellement bénigne ou asymptomatique, mais il existe des formes graves pouvant entraîner une fuite plasmatique, un état de choc, une atteinte des organes (foie, rein, système nerveux central) et une fièvre hémorragique. L’évolution de la dengue peut ainsi être émaillée de complications neurologiques [17,22,25,38]. Les complications neurologiques de la dengue sont rapportées avec une incidence moyenne de 5 % avec des extrêmes entre 0,5 % à 21 %, concernant à la fois le système nerveux périphérique et le système nerveux central (SNC). Elles sont classées en complications directement liées à la neuro invasion par le DENV (encéphalite, méningite, myélite), en complications indirectes comprenant l’encéphalopathie, les syndromes post-infectieux à médiation immunitaire et les manifestations de mécanismes incertains comme les atteintes cérébro-vasculaires [9,14,40,41,48]. La survenue des complications neurologiques constitue un facteur de risque de décès par la dengue, avec une proportion de risque de décès attribuable de 30 à 50 % [2,45]. Ces manifestations et complications neurologiques associées à l’infection à DENV devraient croître avec l’augmentation de l’incidence du DENV dans le monde [13]. Le Burkina Faso a ainsi connu 3 vagues successives d’épidémie de dengue ces 10 dernières années, la dernière flambée épidémique survenue en 2023 a été la plus importante d’Afrique avec 17 125 cas confirmés de dengue et 688 décès [4,37]. Au Burkina Faso, plusieurs travaux sur la dengue ont été publiés [11,37,45], mais aucun n’a porté spécifiquement sur ses atteintes neurologiques. Nous avons donc réalisé le présent travail afin de contribuer à améliorer la base des données sur la dengue en général et le spectre des complications neurologiques de la dengue, en particulier en Afrique subsaharienne. Les objectifs de notre étude étaient de déterminer la fréquence, de décrire le spectre et d’évaluer le pronostic hospitalier des complications neurologiques de la dengue chez les adultes, à travers une étude transversale, hospitalière, multicentrique, rétrospective, durant l’épidémie de dengue de 2023 à Ouagadougou au Burkina Faso. La connaissance de la charge réelle des manifestations neurologiques de l’infection à DENV pourrait contribuer à une meilleure prise en charge et à une amélioration du pronostic des malades.

## Patients et méthodes

Il s’est agi d’une étude transversale à collecte de données rétrospective, descriptive, analytique et multicentrique, ayant concerné les patients adultes hospitalisés pour dengue confirmée dans les CHU Bogodogo, Tengandogo et Yalgado Ouédraogo de Ouagadougou au Burkina Faso, du 1^er^ janvier au 31 décembre 2023, période correspondant à la flambée épidémique de la dengue au Burkina Faso en 2023.

Ont été inclus dans l’étude, les patients âgés de plus de 15 ans, hospitalisés pour dengue confirmée dans les CHU de Ouagadougou (services des urgences, de maladies infectieuses, de médecine interne, de neurologie, de réanimation), et chez qui une ponction lombaire (PL) avec étude cytologique, biochimique et microbiologique du liquide céphalo-rachidien (LCR), a été réalisée. Un cas confirmé de dengue a été défini comme tout cas suspect ou probable de dengue, confirmé en laboratoire par soit une sérologie ELISA positive comportant des IgM, soit une augmentation des titres d’IgG, soit la détection du virus par *Reverse Transcriptase Polymerase Chain Reaction* (RT PCR). Un cas probable de dengue a été défini comme tout cas présentant une fièvre et des symptômes cliniques compatibles avec la mise en évidence d’IgM dans un échantillon, ou d’IgG avec un titre élevé (HI ≥ 1 280), ou un antigène NS1 positif (ELISA ou test rapide).

N’ont pas été inclus dans l’étude les cas suspects mais non confirmés de dengue, les patients qui avaient des dossiers incomplets ou comportant des données insuffisantes ou inexploitables. Les patients qui ont été vus en ambulatoire ou qui présentaient des troubles neurologiques préexistants ont été exclus. En outre, les patients qui avaient une infection à VIH connue ou une goutte épaisse positive pour *Plasmodium falciparum* n’ont pas été inclus.

Les données sociodémographiques, les manifestations cliniques extra neurologiques et neurologiques, les résultats des examens biologiques et de la tomodensitométrie (TDM) cérébrale, ainsi que les données de l’évolution clinique intra hospitalière, ont été recueillis pour chaque patient. Les données ont été collectées à partir des dossiers cliniques des malades, des registres d’hospitalisation et de sorties et des décès. Elles ont été saisies et analysées à l’aide du logiciel STATA 16. Les résultats ont été exprimés en pourcentage et/ ou en moyenne. Afin de déterminer les facteurs indépendamment associés aux décès hospitaliers, nous avons procédé à une analyse univariée puis multivariée avec régression logistique. La survenue ou non de décès hospitaliers était la variable dépendante et les caractéristiques sociodémographiques, cliniques, biologiques et étiologiques, les variables indépendantes. Les variables avec p < 0,20 en analyse univariée ont été introduites dans l’analyse multivariée. Le seuil de significativité était de p < 0,05.

L’étude a été approuvée par les comités d’éthique institutionnels des CHU Tengandogo, Bogodogo et Yalgado Ouédraogo, puis autorisée par les administrations desdits CHU (autorisations n° 2024_037_/MSHP /SG/ CHU-T /DG du 20/02/2024; n° 2024_0295_/MSHP /SG/CHU-YO /DG/ DSP du 29/02/2024; n° 2024 / 000028 / MSHP /SG/CHU-B/DG du 20/02/2024). La confidentialité des données colligées et l’anonymat des patients ont été garantis.

La dengue sévère a été définie par la présence d’un ou plusieurs critères de sévérité établis par l´OMS dans sa classification de 2009 :

1. fuite plasmatique : hémoconcentration (hé-matocrite > 45 %) et/ou état de choc et/ou épanchement liquidien des séreuses;
2. hémorragie sévère : hémorragie diffuse et/ou abondante avec déglobulisation nécessitant une transfusion sanguine;
3. complications organiques : transaminases (ALAT ou ASAT) > 1 000 U/L et/ou atteinte du SNC et/ou thrombopénie sévère < 20 000 éléments/mm^3^ et/ou insuffisance rénale aiguë (créatininémie > 176,8 micromoles/l selon la classification RIFLE) [35].

Chez un patient diagnostiqué pour une dengue confirmée, les définitions de complications neurologiques suivantes ont été appliquées [9,14,33] :

- Encéphalopathie de la dengue. Signes d’atteinte encéphalique, associés à la présence d’au moins une des complications liées à la dengue : insuffisance hépatique, acidose métabolique, insuffisance rénale aiguë, hyponatrémie sévère (natrémie < 120 mmol/l), état de choc prolongé, coagulation intravasculaire disséminée, hémorragie cérébrale, œdème cérébral à la TDM cérébrale; LCR normal (cytologie, protéinorachie, étude microbiologique).
- Encéphalite à DENV probable. Signes cliniques d’atteinte encéphalique, associés à une pléïocytose du LCR et à une protéinorachie élevée en l’absence d’autres agents pathogènes neuro-invasifs (bactériologie, parasitologie, mycologie, à l’examen direct ou après culture, normales).
- Accident vasculaire cérébral (AVC). Apparition brutale d’un déficit neurologique focal de topographie vasculaire cérébrale avec confirmation de la nature ischémique ou hémorragique par la TDM cérébrale, après exclusion des causes classiques d’AVC.
- Encéphalomyélite aiguë disséminée. Évoquée devant l’apparition de signes cliniques d’atteinte encéphalique et/ou médullaire, confirmée par l’imagerie par résonnance magnétique (IRM) encéphalique et médullaire.
- Autres atteintes du système nerveux central (SNC). Apparition de signes d’atteinte du SNC, confirmation par la TDM et/ou l’étude du LCR.
- Atteintes du système nerveux périphérique. Apparition d’un syndrome neurogène périphérique chez un patient ayant une dengue confirmée avec confirmation par un élec-troneuromyogramme (ENMG) et une dissociation albumino-cytologique du LCR (syndrome de Guillain-Barré).
- Atteintes musculaires. Apparition d’un syndrome myogène confirmée par une élévation des enzymes musculaires et/ou par un syndrome myogène à l’ENMG.

## Résultats

En tout, 1 057 patients ont été hospitalisés pour cas de dengue confirmée du 1^er^ janvier au 31 décembre 2023 dans nos sites d’étude : 76 cas confirmés par une sérologie ELISA positive à IgM (7,2 %), 309 cas confirmés par une détection du virus par RT-PCR (29,2 %) et 672 cas fortement présomptifs (augmentation du titre des IgG + antigène NS1 positif (ELISA ou test rapide) (63,6 %). Parmi ces cas, 116 ont présenté des complications neurologiques, soit une prévalence de 11 %. Il s’agissait pour tous de cas confirmés de dengue soit par une sérologie ELISA positive des IgM avec 23 cas (19,8 %) soit par la RT-PCR avec 93 cas (80,2 %). La moyenne d’âge des patients était de 47,6 ans ± 1,94 (extrêmes 16 ans et 95 ans). La tranche d’âge de 20-50 ans comportait 49 patients (42,2 %), le genre féminin 60 patientes (51,7 %). L’hypertension artérielle (HTA) avec 33 cas (28,4 %), le diabète type 2 avec 13 cas (11,2 %) et la drépanocytose avec 7 cas (6 %), étaient les comorbidités les plus fréquentes (Tableau I).

**Tableau I:** Répartition des patients hospitalisés pour dengue ayant des complications neurologiques selon les données sociodémographiques et les comorbidités

Caractéritique	Effectif (n = 116)	Pourcentage
Âge		
≤ 50 ans	62	53,4
50-80 ans	47	40,5
≥ 80 ans	7	6,0
Genre		
féminin	60	51,7
masculin	56	48,3
Antécédents Comorbidités		
HTA	33	28,4
diabète type 2	13	11,2
drépanocytose (SS, SC, CC)	7	6,0
asthme	4	3,4
séquelles d’AVC	3	2,6
ulcère gastro-duodénal	2	1,7
autres	4	3,4

Les motifs d’hospitalisation étaient dominés par les troubles de la conscience avec 50 cas (43,1 %), les signes digestifs sévères avec 35 cas (30,2 %), les signes hémorragiques avec 26 cas (22,4 %), les crises convulsives répétitives avec 22 cas (19 %) et la détresse respiratoire avec 21 cas (18,1 %) (Tableau II).

**Tableau II:** Répartition des patients avec complications neurologiques de la dengue selon le motif d’hospitalisation

Motif d’hospitalisation	Effectif (n = 116)	Pourcentage (%)
Syndrome infectieux sévère persistant	18	15,5
Signes hémorragiques	26	22,4
Signes digestifs sévères	35	30,2
Agitation psychomotrice	17	14,6
Troubles de la conscience	50	43,1
Altération de l’état général	20	17,2
Crises convulsives répétitives	22	19
Perte de connaissance transitoire	4	3,4
Détresse respiratoire	21	18,1
Signes neurologiques focaux	11	9,5

Le délai moyen entre le début de la maladie et l’admission en hospitalisation était de 12,6 jours ± 5,2 (6 – 21 jours).

À l’examen clinique, les signes infectieux, les signes algiques et les signes digestifs, avec respectivement 114 cas (98,3 %), 104 cas (89,7 %) et 49 cas (42,2 %), étaient les signes d’atteintes extra neurologiques les plus fréquemment retrouvés. Les troubles de la conscience avec 98 cas (84,5 %), les crises d’épilepsie avec 24 cas (20,7 %), les signes neurologiques de focalisation avec 19 cas (16,4 %) et les troubles psycho-comportementaux avec 17 cas (14,6 %) étaient les signes cliniques neurologiques les plus fréquemment rencontrés (Tableau III).

**Tableau III:** Répartition des patients hospitalisés pour dengue ayant présenté des complications neurologiques, selon les signes d’atteinte extra neurologiques et les signes d’atteinte du système nerveux (n = 116)

Signes d’atteinte des autres appareils et du système nerveux	Effectif (n = 116)	Pourcentage (%)
Signes infectieux	114	98,3
Signes algiques	104	89,7
Signes digestifs	49	42,2
Syndrome hémorragique	26	22,4
Altération de l’état général	24	20,7
Épanchement pleural et/ou ascite	21	18,1
État de choc	7	6
Signes neurologiques		
troubles de conscience	98	84,5
Glasgow ≤ 8	31	26,7
Glasgow [9-14]	67	57,8
crises épileptiques	24	20,7
troubles psycho-comportementaux	17	14,6
Signes neurologiques de focalisation	19	16,4
hémiplégie/hémiparésie/tétraparésie	19	16,4
aphasie d’expression	2	1,7
syndrome frontal	1	0,9
syndrome neurogène périphérique	1	0,9
syndrome méningé	3	2,6
syndrome d’hypertension intracrânienne	1	0,9

Sur le plan biologique nous avons observé un syndrome de cytolyse hépatique aiguë, une insuffisance rénale aiguë, une thrombopénie, dans respectivement 76 cas (65,5 %), 54 cas (46,5 %) et 44 cas (37,9 %) (Tableau IV).

**Tableau IV:** Répartition des patients hospitalisés pour dengue ayant présenté des complications neurologiques, selon les anomalies biologiques et à la TDM cérébrale (n = 116)

Anomalies biologiques	Effectif (n = 116)	Pourcentage (%)
Anémie	40	34,5
Hyperleucocytose	46	39,6
Leucopénie	6	5,2
Thrombopénie	44	37,9
Hématocrite augmenté	40	34,5
Insuffisance rénale aiguë	54	46,5
Cytolyse hépatique aiguë	76	65,5
Hyperglycémie	22	19
CR Paugmentée	59	50,9
Hypocalcémie	41	35,3
Hyponatrémie	31	26,7
Hypernatrémie	2	1,7
Hypokaliémie	18	15,5
Hyperkaliémie	12	10,3
Hypomagnésémie	9	7,7
TDM cérébrale	(n = 23)	
AVC	7	6
ischémique	2	1,7
hémorragie intracérébrale	5	4,3
Encéphalite	1	0,9
Atrophie cortico-sous-corticale	2	1,7

La TDM cérébrale a été réalisée chez 23 patients (19,8 %) en présence de signes d’appel et a retrouvé un AVC chez 7 patients (6 %).

En utilisant la classification des cas cliniques de dengue selon l’OMS [35], parmi les 116 patients hospitalisés pour dengue avec complications neurologiques, 98 (84,5 %) avaient une dengue sévère et 18 patients (15,5 %) une dengue avec signes d’alerte. Parmi les cas cliniques de dengue sévère, les troubles de la vigilance avec 98 cas (84,5 %) et les atteintes sévères d’organes avec 76 cas (65,5 %) étaient les plus fréquemment rencontrés (Tableau V).

**Tableau V:** Répartition des patients hospitalisés pour dengue ayant des complications neurologiques selon la classification des cas cliniques de dengue par l’OMS en 2009 (n = 116)

Classification des cas cliniques de dengue selon l’OMS	Effectif (n = 116)	Pourcentage (%)
Dengue avec signes d’alerte	18	15,5
Dengue sévère	98	84,5
troubles de la vigilance	98	84,5
syndrome de fuite plasmatique	40	34,5
avec détresse respiratoire (épanchement pleural, ascite, œdèmes)	24	20,7
avec état de choc	7	6
hémorragie sévère ayant nécessité une transfusion sanguine	12	10,3
atteinte sévère d’organes (isolées ou associées)	76	65,5
cytolyse hépatique sévère (ASAT ou ALAT >10 000 U/l)	32	27,6
insuffisance rénale aiguë sévère (créatininémie > 300 µmol/l)	28	24,1
thrombopénie sévère	37	31,9

Une ponction lombaire avec étude du LCR et un ENMG ont permis de confirmer un syndrome de Guillain-Barré (SGB) à forme sensitivomotrice démyélinisante chez un patient qui avait présenté un syndrome neurogène périphérique sensitivomoteur d’évolution ascendante.

Le spectre clinique des complications neurologiques chez les patients hospitalisés pour dengue était représenté par l’encéphalopathie métabolique avec 99 cas (9,4 %), l’encéphalite à DENV probable avec 9 cas (0,8 %), les AVC avec 7 cas (0,7 %) et le SGB avec 1 cas (0,1 %) (Tableau VI).

**Tableau VI:** Spectre clinique des complications neurologiques chez les patients hospitalisés pour dengue (n = 1 057) et chez les patients hospitalisés pour dengue avec complications neurologiques (116)

Spectre clinique des complications neurologiques de la dengue	Effectif		Pourcentage (%)
	**116**	**1 057**	
Encéphalopathie métabolique	99	85,3	9,4
Encéphalite à DENV probable	9	7,8	0,8
AVC	7	6,0	0,7
hémorragie intra cérébrale	5	4,3	0,5
ischémique	2	1,7	0,2
Syndrome de Guillain-Barré	1	0,9	0,1

Le service d’accueil des urgences avec 63 patients hospitalisés (54,3 %) et le service de réanimation avec 26 cas (22,4 %), ont été les principaux services d’hospitalisation (Tableau VII).

**Tableau VII:** Répartition des patients avec complications neurologiques selon le service d’hospitalisation directe

Service d’hospitalisation	Effectif (n = 116)	Pourcentage (%)
Service d’accueil des urgences médicales	63	54,3
Service de réanimation	26	22,4
Service des maladies infectieuses	19	16,4
Service de médecine interne ou de neurologie	8	6,9

Le traitement a consisté principalement en l’utilisation d’antalgiques chez 114 patients (98,3%), d’antibiotiques chez 86 patients (74,1%), d’oxygénothérapie chez 58 patients (50%) et de transfusion de culots globulaires ou de plaquettes chez 32 patients (27,6%) (Tableau VIII).

**Tableau VIII:** Répartition des patients hospitalisés pour dengue avec complications neurologiques selon les traitements reçus

Traitements reçus	Effectif (n = 116)	Pourcentage (%)
Antalgiques	114	98,3
Antibiotiques	86	74,1
Oxygénothérapie	58	50,0
Transfusion (culots globulaires ou plaquettaires)	32	27,6
Hémostatiques	25	21,5
Antiémétiques	19	16,4
Anti HTA	18	15,5
Antiépileptiques	17	14,7
Antidiabétiques	17	14,7
Antipsychotiques	9	7,8
Hémodialyse	5	4,3
Antidépresseurs	5	4,3
Antiagrégants plaquettaires	5	4,3
Antiœdémateux	3	2,6
Corticothérapie	2	1,7
Immunoglobulines	1	0,9
Héparinothérapie curative	1	0,9
Antiviraux	1	0,9

La durée moyenne d’hospitalisation a été de 4,5 jours ±0,5 jours. En tout, 80 patients sont décédés à l’hôpital, soit une létalité hospitalière de 69 %. Dix patients (9 %) ont été perdus de vue. Les 26 autres patients ont survécu, parmi lesquels 12 patients gardaient des séquelles neurologiques lors de leur sortie d’hospitalisation.

En analyse bivariée, le coma (p = 0,02), les crises d’épilepsie (p = 0,03), les troubles de la vigilance (p = 0,00), la désaturation (p = 0,00) et l’insuffisance rénale aiguë (p = 0,00)*,* étaient significativement associés au décès hospitalier. En analyse multivariée avec régression logistique pas à pas, l’insuffisance rénale aiguë (OR = 3,49; p = 0,02) était le seul facteur indépendamment associé au décès (Tableau XI).

**Tableau IX:** Résultats de l’analyse univariée puis multivariée pour l’identification des variables sociodémographiques, cliniques et biologiques, significativement associées au décès intra hospitalier

	Analyse univariée			Analyse multivariée		
Variables	Total	Décès	p-value	OR	95% IC	p-value
Âge > 50 ans	116	80	-			
Sexe masculin	56	34	0,07			
Sexe féminin	60	46	0,07			
Antécédents d’HTA	33	24	0,58			
Diabète type 2	13	10	0,51			
Antécédents d’AVC ischémique	3	2	0,93			
Déficit moteur	16	10	0,55			
Signes infectieux	43	33	0,17			
Poussée d’HTA lors de l’admission	28	19	0,88			
Troubles de la vigilance (Glasgow ≤ 14)	98	75	0,00			
Coma (Glasgow ≤ 8)	31	27	0,02	2,49	0,65 – 9,44	0,18
Désaturation (SpO2<95%)	61	50	0,00	2,37	0,41 – 6,76	0,24
Crises épileptiques	24	12	0,03	0,82	0,17 – 3,86	0,80
Insuffisance rénale aiguë	54	47	0,00	3,49	1,19 – 10,27	0,02
Cytolyse hépatique	76	55	0,27			
Hyperglycémie	22	18	0,09			
Thrombopénie	44	32	0,33			
Complication neurologique type encéphalopathie métabolique	99	76	0,00	1,49	0,28 – 7,80	0,64

## Discussion

Les manifestations neurologiques liées aux infections par la dengue sont de plus en plus souvent observées, en particulier au cours des épidémies et représentent un défi pour la pratique médicale, avec une incidence variant entre 0,5 et 20 %, selon les données récentes de la littérature [1,9,13,25,33,38-41,48].

Il est habituel dans la littérature de diviser le spectre des manifestations neurologiques de la dengue en trois catégories :

a) les complications liées au neurotropisme direct du virus de la dengue - encéphalite, méningite, myélite et myosite;
b) les complications systémiques liées à l’infection par la dengue - encéphalopathie, AVC et paralysie hypokaliémique;
c) les complications post-infectieuses à médiation immunitaire, principalement encéphalomyélite aiguë disséminée, SGB et névrite optique [9,14,24,33,38].

L’encéphalite à DENV est une atteinte neurologique fréquente de l’infection par la dengue dans différentes parties du monde [18,24,34,43,44]. Dans notre série, l’encéphalite à DENV n’a cependant été retrouvée que chez 0,8 % des patients hospitalisés pour dengue.

Les critères de diagnostic de l’encéphalite à DENV reposent sur la présence d’une fièvre et de signes aigus d’atteinte encéphalique, associés à la mise en évidence d’anticorps anti-DENV de type IgM ou d’ARN de DENV ou d’antigène NS1 dans le sérum et/ou le LCR, après exclusion d’autres causes d’encéphalite virale et des causes d’encéphalopa-thie [43]. Les examens de tomodensitométrie (CT) et d’imagerie par résonance magnétique (IRM) ne sont pas toujours contributifs car ils peuvent être normaux. Dans notre série, le diagnostic d’encéphalite à DENV a été évoqué en présence de signes d’atteinte encéphalique dans les 30 jours suivant l’installation d’une dengue confirmée par la positivité des anticorps anti-DENV de type IgM ou de l’ARN de DENV ou d’antigène NS1 dans le sérum. Ce diagnostic a été confirmé par la mise en évidence d’un LCR clair avec pléïocytose et hyperprotéinorachie, à l’instar des séries de Soares *et al*. [43] et Carod-Artal *et al*. [9], après exclusion des autres causes d’encéphalites à LCR clair (examen bactériologique, parasitologique et mycologique du LCR, sérologie VIH négative). Les marqueurs de l’infection à DENV dans le LCR (anticorps anti-DENV de type IgM ou ARN de DENV par PCR ou antigène NS1 dans le LCR) n’ont pu être mis en évidence faute de disponibilité de ces examens dans notre contexte. Cependant, une cellularité et une protéinorachie normales du LCR ne suffisent pas toujours à exclure une encéphalite à DENV [31]. Les marqueurs de l’encéphalite à DENV dans le LCR sont indispensables pour le diagnostic de certitude, en particulier des encéphalites à DENV à pléïocytose absente du LCR dont la proportion atteint 20 % des cas [2,7]. La non-disponibilité des méthodes de détection des marqueurs de l’encéphalite à DENV dans le LCR a probablement contribué à leur sous diagnostic dans notre étude. Récemment, Fong *et al*. ont proposé des critères diagnostics plus contraignants : exigence de détection du DENV dans le LCR et/ou le tissu cérébral pour le diagnostic définitif de l’encéphalite à DENV et mise en évidence d’IgM anti-DENV dans le LCR pour le diagnostic d’encéphalite à DENV probable [14]. Les autres infections neuro invasives du SNC à DENV telles la méningite et la myélite à DENV sont plus rarement rapportées comme en atteste notre série, où aucun de ces cas n’a été retrouvé [39,43,49].

L’encéphalopathie associée à la dengue est décrite comme la manifestation neurologique la plus fréquente de la dengue [9,33,39]. Son diagnostic est évoqué devant des signes de dysfonctionnement encéphalique [9,39]. Il est confirmé par une étude du LCR normale (cytologie, protéino-rachie, glycorachie) [9]. L’encéphalopathie liée à la dengue résulte de facteurs étiologiques multiples, notamment un œdème cérébral, une hypoxie et/ ou une hémorragie cérébrales et des dysfonctionnements systémiques associés comme un état de choc prolongé, une hyponatrémie profonde, une insuffisance hépatique, une insuffisance rénale, une acidose métabolique, une coagulation intra vasculaire disséminée ou une libération de substances toxiques (orage cytokinique) associée à un syndrome de perméabilité vasculaire [9,16,21,24,33].

L’incidence de l’encéphalopathie liée à la dengue varie d’une étude à l’autre en fonction du type de population étudiée (adultes ou enfants) [9,24]. Dans notre étude, nous avons enregistré une encéphalopathie chez 9,4 % des cas de dengue hospitalisés, *versus* 2,5 % des enfants hospitalisés pour dengue en Thaïlande [36], 0,8 % des patients hospitalisés pour dengue en Inde [24] et 0,5 % des patients atteints de dengue confirmée au Vietnam [8]. L’incidence élevée de l’encéphalopathie associée à la dengue dans notre contexte pourrait s’expliquer par un biais de recrutement des cas cliniques de dengue sévère (84,5 %) dans les hôpitaux de 3^e^ niveau de référence à Ouagadougou dans un contexte épidémique, l’admission tardive des patients au stade des complications souvent intriquées et une défaillance polyviscérale. En effet, dans notre étude, les complications neurologiques coexistaient avec une atteinte sévère d’organes (65 %), un syndrome de fuite plasmatique (34,5 %), une hémorragie sévère avec nécessité de transfusion sanguine (10,3 %).

Le SGB est rapporté comme étant la complication post-infectieuse à médiation immunitaire la plus fréquente [27]. Dans les séries de Verma *et al*. en Inde [47] et de Jackson *et al*. à la Jamaïque [18], le SGB représentait respectivement 11,5 % et 3,7 % des patients ayant des complications neurologiques de la dengue, alors que dans notre étude le SGB ne représentait que 0,9 % des complications neurologiques de la dengue. La faible proportion des cas de SGB observée dans notre série pourrait être due à la courte durée d’hospitalisation qui était en moyenne de 4,5 jours, alors que les délais d’apparition du SGB post dengue s’échelonnent de 5 à 15 jours [15,46]. Le SGB post dengue est caractérisé sur le plan électrophysiologique par une prédominance des formes démyélinisante

[9] et sensitivomotrice axonale [9,15], ce qui a été retrouvé dans notre étude. Le mécanisme du SGB associé au DENV est le mimétisme moléculaire entre les protéines humaines relative au SGB et la polyprotéine du DENV [50,51]. Les auteurs recommandent la recherche systématique d’une infection récente à DENV dans les cas de SGB en zone endémique [47].

Les autres syndromes post infectieux de la dengue à médiation immunitaire prouvée ou fortement suspectée rapportés dans la littérature n’ont pas été retrouvés dans notre série, faute de disponibilité de moyens diagnostiques appropriés, notamment l’IRM encéphalique et la PCR du DENV dans le LCR. Il s’agit essentiellement de l’encéphalomyélite aiguë disséminée et de la névrite optique [14,20]. D’autres syndromes associés à la dengue selon un mécanisme encore incertain n’ont également pas été retrouvés dans notre série : cérébellite [12], myélite aiguë transverse [3], myosite [31].

Des complications cérébro-vasculaires à type d’hémorragies intracrâniennes ou d’AVC ischémiques ont été signalées au cours de la dengue, avec de faibles incidences. Les hémorragies intracrâniennes plus fréquemment rapportées, concernent les hémorragies intra parenchymateuses, les hémorragies sous durales ou sous arachnoïdiennes [9,24,42]. Dans notre série, 0,5 % des patients hospitalisés pour dengue ont présenté une hémorragie intra parenchymateuse cérébrale. Des fréquences quasi similaires ont été rapportées dans d’autres études hospitalières : 0,1 à 0,3 % et 1,8 % des cas hospitalisés pour dengue, respectivement en Inde [24,30] et au Pakistan [32]. Les données actuelles suggèrent que les hémorragies intracrâniennes compliquant la dengue proviendraient d’une surproduction de cytokines pro inflammatoires responsables des lésions des cellules endothéliales à médiation immunitaire, d’une vasculopathie, de la thrombopénie et d’un dysfonctionnement plaquettaire, responsables d’une diathèse hémorragique [5,24].

De rares cas d’AVC ischémiques compliquant une dengue ont été décrits dans la littérature [24,47] et dans notre série. Les mécanismes possibles incluent un dérèglement de l’hémostase, une grave déplétion volumique, une hypotension, une méningovascularite ou une myocardite [24]. L’intégration des examens de diagnostic de la dengue dans le bilan étiologique des AVC en zone d’endémie de la dengue pourrait contribuer à une meilleure caractérisation de ses complications cérébrovasculaires.

Dans les pays asiatiques et sud-américains, alors que la mortalité due à l’infection par la dengue se situe entre 3 % et 5 % [39], la létalité des patients qui développent une complication neurologique associée de la dengue varie entre 5 % et 30 % [17,28,32,40]. Au Burkina Faso, une létalité intra hospitalière de 7,8 % a été rapportée chez des patients hospitalisés pour dengue sévère en 2016, alors que les complications neurologiques représentaient 63,4 % des causes de décès [45]. De même, dans une autre étude rétrospective réalisée au Brésil, 48,8 % des décès dus à la dengue étaient attribuables aux complications neurologiques [2]. Dans une étude réalisée au Pakistan, la survenue de complications neurologiques chez les patients hospitalisés pour dengue, constituait un important facteur prédictif de décès [32].

Avec des taux de létalité de la dengue et des cas de dengue avec complications neurologiques, respectivement de 10 % et 69 %, notre étude confirme la forte létalité attribuable aux complications neurologiques de la dengue, en particulier dans notre contexte. Plusieurs facteurs pourraient expliquer cette situation malheureuse. On peut citer l’admission et la prise en charge tardive des patients au stade des complications parfois gravissimes menaçant à brève échéance le pronostic vital (altération de la conscience voire coma, état de mal épileptique dépassé, détresse respiratoire, hémorragie massive, coagulation intravasculaire disséminée, etc.) nécessitant une prise en charge spécialisée en urgence. Ainsi, le délai d’admission moyen en hospitalisation de nos patients était de 12 jours, alors que les complications de la dengue apparaissent vers le 7^e^ jour après le début de la maladie.

Parmi les causes de ces retards d’admission et de prise en charge des cas de dengue compliqués, on peut citer :

a) la fréquence élevée de la pratique de l’automédication par les populations, les patients ne consultant qu’à la phase des complications graves [50];
b) l’insuffisance d’utilisation systématique des tests de diagnostic biologique du paludisme (goutte épaisse et frottis mince) et de la dengue (tests de détection rapide de l’antigène NS1 de la dengue, des anticorps IgG et des anticorps IgM). Dans un contexte de double endémie palustre et de la dengue au Burkina Faso, cette insuffisance entretient la confusion ou l’incertitude diagnostique, responsable d’un retard diagnostique et de prise en charge adéquate [6]. Il faut également ajouter la non-disponibilité des tests de diagnostic de la dengue en 2023, du fait d’une pénurie de tests sur le marché mondial, en particulier en Afrique et surtout dans la région du Sahel, correspondant à la période de l’épidémie de dengue au Burkina Faso [29];
c) la disponibilité insuffisante des lits de réanimation dans les CHU de Ouagadougou, associée à leur inaccessibilité du fait des difficultés financières des familles à supporter les charges liées à l’hospitalisation et aux soins en l’absence d’assurance maladie universelle au Burkina Faso, classé comme pays à faibles revenus [19]. Ainsi seuls 22 % de nos patients ont été transférés en réanimation médicale contre 80 % qui avaient cette indication;
d) enfin les difficultés de la prise en charge médicale des formes compliquées du fait de leurs complexités, la non-disponibilité de certains moyens thérapeutiques, l’absence ou la méconnaissance des protocoles de soins de la dengue compliquée.

Afin d’inverser cette tendance, nous suggérons aux autorités du ministère de la santé du Burkina Faso de :

a) poursuivre et intensifier la prévention primaire de la dengue à travers la lutte antivectorielle;
b) accélérer l’accessibilité et la disponibilité des tests de diagnostic rapide de la dengue (et du paludisme);
c) produire et disséminer des directives réglementaires et des protocoles clairs de prise en charge des cas de dengue au niveau communautaire et aux différents niveaux de notre pyramide sanitaire nationale;
d) intégrer les moyens du traitement de la dengue parmi les médicaments et consommables essentiels.

Dans notre étude, l’insuffisance rénale aiguë a été identifiée comme un facteur de risque indépendant de la mortalité intra hospitalière des patients hospitalisés pour dengue avec complications neurologiques (OR = 3,49; p = 0,02); un constat similaire a été fait dans d’autres études [26,28]. En effet, l’insuffisance rénale aiguë (IRA) est rapportée comme une complication fréquente et sévère de la dengue, associée à une lourde létalité qui lui est attribuable [11,34,45]. L’IRA contribue ainsi fortement à l’augmentation de l’incidence et de la létalité des complications neurologiques de la dengue, notamment par l’encéphalopathie urémique qu’elle provoque [11,28].

## Conclusion

Notre étude consacrée à l’épidémie de dengue survenue en 2023 au Burkina Faso a permis de montrer que plus d’un patient sur 10 adultes hospitalisés a présenté des complications neurologiques, dominées par des cas d’encéphalopathie métabolique, suivis de cas d’encéphalite à DENV probable et de rares cas d’AVC et de SGB. Leur évolution clinique a été grevée d’une surmortalité d’environ 7 cas sur 10, favorisée par la coexistence d’une IRA. Afin d’inverser cette tendance, il faut organiser une prise en charge hospitalière précoce et adaptée dans des unités de soins intensifs équipées pour les cas de dengue sévère. Il faut également mener des recherches opérationnelles afin d’identifier, prévenir et prendre en charge les facteurs prédictifs de survenue des complications de la dengue, notamment neurologiques. Et ceci sans omettre la prévention par l’intensification de la lutte anti vectorielle, l’amélioration de la disponibilité et de l’accessibilité des tests de diagnostic rapide de la dengue, et l’application des directives et protocoles nationaux de prise en charge à tous les échelons de notre pyramide sanitaire.

## Financement

L’étude n’a bénéficié d’aucun financement.

## Contribution des auteurs et autrices

LOMPO Djingri Labodi : conception de l’étude, recherche documentaire, recueil des données, élaboration du protocole de l’étude, analyse des données, rédaction du manuscrit. KAGAMBEGA D Sylvie : recueil des données, analyse des données, recherche documentaire, rédaction du manuscrit. OUATTARA Souleymane : recueil des données, révision et validation du protocole, révision du manuscrit. GNAMPA Melody Zeinab : recueil des données. KERE M Fabienne Y : recueil des données. NAPON Christian : validation du protocole de l’étude, supervision de l’étude. MILLOGO Athanase : validation du protocole de l’étude, supervision de l’étude.

## Déclaration de liens d’intérêt

Aucun lien d’intérêt n’a été déclaré.

## References

[1] Aradhya GH, Kumar S (2015 Oct). Central nervous system manifestations and its outcome in Dengue fever. RGUHS Med Sciences..

[2] Araújo FM, Araújo MS, Nogueira RM, Brilhante RS, Oliveira DN, Rocha MF, Cordeiro RA, Araújo RM, Sidrim JJ (2012 Mar 6). Central nervous system involvement in dengue: a study in fatal cases from a dengue endemic area. Neurology..

[3] Badat N, Abdulhussein D, Oligbu P, Ojubolamo O, Oligbu G (2018 Dec 23). Risk of Transverse Myelitis Following Dengue Infection: A Systematic Review of the Literature. Pharmacy (Basel)..

[4] Bangoura ST, Keita AK, Diaby M, Sidibé S, Le-Marcis F, Camara SC, Maltais S, Kadio KJJO, D’Ortenzio E, Camara A, Delaporte E, Delamou A, Vanhems P, Ottmann M, Khanafer N, Touré A (2026 Feb). Arbovirus Epidemics as Global Health Imperative, Africa, 2023. Emerg Infect Dis..

[5] Basu A, Chaturvedi UC (2008 Aug). Vascular endothelium: the battlefield of dengue viruses. FEMS Immunol Med Microbiol..

[6] Bisoffi Z, Sirima SB, Menten J, Pattaro C, Angheben A, Gobbi F, Tinto H, Lodesani C, Neya B, Gobbo M, Van den Ende J (2010 Jul 7). Accuracy of a rapid diagnostic test on the diagnosis of malaria infection and of malaria-attributable fever during low and high transmission season in Burkina Faso. Malar J..

[7] Borawake K, Prayag P, Wagh A, Dole S (2011 Jul). Dengue encephalitis. Indian J Crit Care Med..

[8] Cam BV, Fonsmark L, Hue NB, Phuong NT, Poulsen A, Heegaard ED (2001 Dec). Prospective case-control study of encephalopathy in children with dengue hemorrhagic fever. Am J Trop Med Hyg..

[9] Carod-Artal FJ, Wichmann O, Farrar J, Gascón J (2013 Sep). Neurological complications of dengue virus infection. Lancet Neurol..

[10] Colón-González FJ, Sewe MO, Tompkins AM, Sjödin H, Casallas A, Rocklöv J, Caminade C, Lowe R (2021 Jul). Projecting the risk of mosquito-borne diseases in a warmer and more populated world: a multi-model, multi-scenario intercomparison modelling study. Lancet Planet Health..

[11] Coulibaly G, Lengani HYA, Sondo KA, Konvolbo HP, Diendéré ÉA, Nitiéma IJ, Karambiri AR, Sanou G, Lengani A (2020 Feb). Épidémiologie de l’insuffisance rénale aiguë au cours de la dengue dans la ville de Ouagadougou. Nephrol Ther..

[12] de Holanda AC, Maranhão E, Van Der Linden Ferreira Silva L, Bezerra MER, de Melo ES (2022 Jun). Dengue fever presenting as acute cerebellar ataxia: Case report and literature review. J Neurovirol..

[13] Domingues RB, Kuster GW, Onuki-Castro FL, Souza VA, Levi JE, Pannuti CS (2008 Apr 15). Involvement of the central nervous system in patients with dengue virus infection. J Neurol Sci..

[14] Fong SL, Wong KT, Tan CT (2024 Mar 1). Dengue virus infection and neurological manifestations: an update. Brain..

[15] Fragoso YD, Gomes S, Brooks JB, Matta AP, Ruocco HH, Tauil CB, Sousa NA, Spessotto CV, Grippe T (2016 Dec). Guillain-Barré syndrome and dengue fever: report on ten new cases in Brazil. Arq Neuropsiquiatr..

[16] Halstead SB, Crowe J. E., Boraschi D., Rappuoli R. (2015). Dengue Antibody-Dependent Enhancement: Knowns and Unknowns. Antibodies for Infectious Diseases.

[17] Hopkins HK, Traverse EM, Barr KL (2022 Feb 9). Viral Parkinsonism: An underdiagnosed neurological complication of Dengue virus infection. PLoS Negl Trop Dis..

[18] Jackson ST, Mullings A, Bennett F, Khan C, Gordon-Strachan G, Rhoden T (2008 Sep). Dengue infection in patients presenting with neurological manifestations in a dengue endemic population. West Indian Med J..

[19] Kagambega MT (2020). (2020). Les obstacles à l’effectivité de l’assurance maladie universelle au Burkina Faso.

[20] Kamel MG, Nam NT, Han NHB, El-Shabouny AE, Makram AM, Abd-Elhay FA, Dang TN, Hieu NLT, Huong VTQ, Tung TH, Hirayama K, Huy NT (2017 Jun 30). Post-dengue acute disseminated encephalomyelitis: A case report and meta-analysis. PLoS Negl Trop Dis..

[21] Katzelnick LC, Gresh L, Halloran ME, Mercado JC, Kuan G, Gordon A, Balmaseda A, Harris E (2017 Nov 17). Antibody-dependent enhancement of severe dengue disease in humans. Science..

[22] Koshy JM, Joseph DM, John M, Mani A, Malhotra N, Abraham GM, Pandian J (2012 Oct). Spectrum of neurological manifestations in dengue virus infection in Northwest India. Trop Doct..

[23] Kraemer MUG, Reiner RC, Brady OJ, Messina JP, Gilbert M, Pigott DM, Yi D, Johnson K, Earl L, Marczak LB, Shirude S, Davis Weaver N, Bisanzio D, Perkins TA, Lai S, Lu X, Jones P, Coelho GE, Carvalho RG, Van Bortel W, Marsboom C, Hendrickx G, Schaffner F, Moore CG, Nax HH, Bengtsson L, Wetter E, Tatem AJ, Brownstein JS, Smith DL, Lambrechts L, Cauchemez S, Linard C, Faria NR, Pybus OG, Scott TW, Liu Q, Yu H, Wint GRW, Hay SI, Golding N (2019). Past and future spread of the arbovirus vectors Aedes aegypti and Aedes albopictus. Nat Microbiol..

[24] Kulkarni R, Pujari S, Gupta D (2021 Sep-Oct). Neurological Manifestations of Dengue Fever. Ann Indian Acad Neurol..

[25] Kumar P, Wei Gao P, Veeresha P, Erturk VS, Prakasha DG, Baskonus H M (2024). Solution of a dengue fever model via fractional natural decomposition and modified predictor–corrector methods. International Journal of Modeling, Simulation, and Scientific Computing..

[26] Kuo MC, Lu PL, Chang JM, Lin MY, Tsai JJ, Chen YH, Chang K, Chen HC, Hwang SJ (2008 Sep). Impact of renal failure on the outcome of dengue viral infection. Clin J Am Soc Nephrol..

[27] Leung C (2020 Dec). A lesson learnt from the emergence of Zika virus: What flaviviruses can trigger Guillain-Barré syndrome?. J Med Virol..

[28] Mallhi TH, Khan AH, Sarriff A, Adnan AS, Khan YH, Jummaat F (2016 Feb). Defining acute kidney injury in dengue viral infection by conventional and novel classification systems (AKIN and RIFLE): a comparative analysis. Postgrad Med J..

[29] Manigart O, Ouedraogo I, Ouedraogo HS, Sow A, Lokossou VK (2024 Feb 3). Dengue epidemic in Burkina Faso: how can the response improve?. Lancet..

[30] Mathew S, Pandian JD (2010 May). Stroke in patients with dengue. J Stroke Cerebrovasc Dis..

[31] Misra UK, Kalita J, Maurya PK, Kumar P, Shankar SK, Mahadevan A (2012 Apr). Dengue-associated transient muscle dysfunction: clinical, electromyography and histopathological changes. Infection..

[32] Mohammed WW, Ahmad H, Hamza AE, Aly ES, El-Morshedy M, Elabbasy EM (2021). The exact solutions of the stochastic Ginzburg–Landau equation. Results in Physics..

[33] Murthy JM (2010 Jul-Aug). Neurological complication of dengue infection. Neurol India..

[34] Neeraja M, Iakshmi V, Teja VD, Lavanya V, Priyanka EN, Subhada K, Parida MM, Dash PK, Sharma S, Rao PV, Reddy G (2014 Jul). Unusual and rare manifestations of dengue during a dengue outbreak in a tertiary care hospital in South India. Arch Virol..

[35] Organisation mondiale de la santé (OMS) (2009). Dengue: Guidelines for Diagnosis, Treatment, Prevention and Control – New Edition.

[36] Pancharoen C, Kulwichit W, Tantawichien T, Thisyakorn U, Thisyakorn C (2002 Jun). Dengue infection: a global concern. J Med Assoc Thai..

[37] Poda A, Da L, Somé D, Zoungrana J, Savadogo M, Diallo I, Diendéré A, Ouédraogo A, Kagoné T, Diao R (2026). Deadly dengue epidemic outbreak in Burkina Faso in 2023. Int J Infect Dis..

[38] Prabhat N, Ray S, Chakravarty K, Kathuria H, Saravana S, Singh D, Rebello A, Lakhanpal V, Goyal MK, Lal V (2020 Dec). Atypical neurological manifestations of dengue fever: a case series and mini review. Postgrad Med J..

[39] Puccioni-Sohler M, Rosadas C, Cabral-Castro MJ (2013 Sep). Neurological complications in dengue infection: a review for clinical practice. Arq Neuropsiquiatr..

[40] Sahu R, Verma R, Jain A, Garg RK, Singh MK, Malhotra HS, Sharma PK, Parihar A (2014 Oct 28). Neurologic complications in dengue virus infection: a prospective cohort study. Neurology..

[41] Saini L, Chakrabarty B, Pastel H, Israni A, Kumar A, Gulati S (2017 May-Jun). Dengue fever triggering hemiconvulsion hemiplegia epilepsy in a child. Neurol India..

[42] Seet RC, Lim EC (2007 Aug). Dysarthria-clumsy hand syndrome associated with dengue type-2 infection. J Neurol..

[43] Soares CN, Cabral-Castro MJ, Peralta JM, de Freitas MR, Zalis M, Puccioni-Sohler M (2011 Apr 15). Review of the etiologies of viral meningitis and encephalitis in a dengue endemic region. J Neurol Sci..

[44] Solomon T, Dung NM, Vaughn DW, Kneen R, Thao LT, Raengsakulrach B, Loan HT, Day NP, Farrar J, Myint KS, Warrell MJ, James WS, Nisalak A, White NJ (2000 Mar 25). Neurological manifestations of dengue infection. Lancet..

[45] Sondo KA, Gnamou A, Diallo I, Ka D, Zoungrana J, Diendéré EA, Fortes L, Poda A, Ly D, Ouédraogo AG (2022 Mar). Étude descriptive des complications de la dengue au cours de la flambée de 2016 à Ouagadougou au Burkina Faso. PAMJ-One Health..

[46] Tan CY, Razali SNO, Goh KJ, Sam IC, Shahrizaila N (2019 Nov). Association of dengue infection and Guillain-Barré syndrome in Malaysia. J Neurol Neurosurg Psychiatry..

[47] Verma R, Sharma P, Garg RK, Atam V, Singh MK, Mehrotra HS (2011 Oct). Neurological complications of dengue fever: Experience from a tertiary center of north India. Ann Indian Acad Neurol..

[48] Waggoner JJ, Gresh L, Vargas MJ, Ballesteros G, Tellez Y, Soda KJ, Sahoo MK, Nuñez A, Balmaseda A, Harris E, Pinsky BA (2016 Dec 15). Viremia and Clinical Presentation in Nicaraguan Patients Infected With Zika Virus, Chikungunya Virus, and Dengue Virus. Clin Infect Dis..

[49] Wasay M, Channa R, Jumani M, Shabbir G, Azeemuddin M, Zafar A (2008 Jun). Encephalitis and myelitis associated with dengue viral infection clinical and neuroimaging features. Clin Neurol Neurosurg..

[50] Yameogo RA, Bere NLU, Zabsonre P, Meda N (2024 dec 14). L’automédication à l’ère de l’internet dans la ville de Ouagadougou: une enquête menée auprès des pharmacies. J Afr Technol Pharm Biopharm..

[51] Yuki N (2001 Aug). Infectious origins of, and molecular mimicry in, Guillain-Barré and Fisher syndromes. Lancet Infect Dis..

