# Supplementary material for: Neurological complications of dengue fever in adults at Ouagadougou university hospitals during the 2023 epidemic in Burkina Faso
Source: Med Trop Sante Int. 2026 Jan 7;6(1):mtsi.v6i1.2026.794. [Article in French] doi: 10.48327/mtsi.v6i1.2026.794 (PMC13196132; doi:10.48327/mtsi.v6i1.2026.794)
Supplement: Related texts

# Research article: Neurological complications of dengue fever in adults at Ouagadougou university hospitals during the 2023 epidemic in Burkina Faso

## Introduction

Dengue is an infectious disease caused by *Orthoflavivirus denguei* (Dengue virus, DENV), transmitted by *Aedes aegypti* and *Ae. albopictus* [23]. Endemic to tropical and subtropical regions, dengue is widespread and has a steadily increasing annual incidence worldwide, reaching 100 to 400 million infections per year. It is estimated that an additional 4.7 billion people will be at risk globally by 2070. In sub-Saharan Africa, particularly in Burkina Faso, dengue is endemic and epidemic. It is spreading rapidly due to climate change, the geographical expansion of vectors, increased human mobility, rapid and unplanned urbanization, a lack of sustainable vector control, co-circulation and emergence of several serotypes (DENV 1 to 4) [10,14].

Dengue typically presents as a mild or asymptomatic febrile illness, but severe forms can lead to plasma leakage, shock, and organ damage to the liver, kidneys, and central nervous system, as well as hemorrhagic fever. Thus, the course of dengue can be marked by neurological complications [17,22,25,38]. These complications are reported with an average incidence of 5%, ranging from 0.5% to 21%, and affect both the peripheral and central nervous systems (CNS). These complications are classified as either directly related to DENV neuroinvasion (e.g., encephalitis, meningitis, and myelitis) or indirectly related (e.g., encephalopathy, post-infectious immune-mediated syndromes, and manifestations of uncertain mechanisms, such as cerebrovascular damage) [9,14,40,41,48]. The occurrence of these complications is a risk factor for dengue-related death, with an attributable risk ranging from 30% to 50% [2,45]. These neurological manifestations and complications associated with DENV infection are expected to increase with the rise in DENV incidence worldwide [13]. Burkina Faso has experienced three successive waves of dengue epidemics over the past 10 years. The latest outbreak, in 2023, was the largest in Africa, with 17,125 confirmed dengue cases and 688 deaths [4,37]. Although several studies on dengue have been published in Burkina Faso, none have focused specifically on its neurological effects [11,37,45]. Therefore, we conducted this study to improve the database on dengue in general and the spectrum of its neurological complications in particular in sub-Saharan Africa. Our study aimed to determine the frequency and describe the spectrum of dengue-related neurological complications in adults. We also sought to assess the hospital prognosis of these complications through a cross-sectional, hospital-based, multi-center, retrospective study conducted during the 2023 dengue epidemic in Ouagadougou, Burkina Faso. Understanding the extent of neurological manifestations of dengue virus (DENV) infection could lead to better management and improved prognoses for patients.

## Patients and methods

This cross-sectional, retrospective, descriptive, and analytical multicenter study involved adult patients hospitalized with confirmed dengue fever at Bogodogo, Tengandogo, and Yalgado Ouédraogo university hospitals in Ouagadougou, Burkina Faso, from January 1 to December 31, 2023. This time period corresponded with the 2023 dengue epidemic in Burkina Faso.

The study included patients over the age of 15 hospitalized for confirmed dengue fever in the emergency, infectious diseases, internal medicine, neurology, and intensive care departments of the university hospitals in Ouagadougou who underwent lumbar puncture (LP) with cytological, biochemical, and microbiological examination of the cerebrospinal fluid (CSF). A confirmed case of dengue was defined as a suspected or probable case confirmed by a positive ELISA serology test for IgM, an increase in IgG titers, or the detection of the virus by reverse transcriptase polymerase chain reaction (RT-PCR). A probable case of dengue was defined as a case presenting with fever and clinical symptoms consistent with dengue, or the detection of IgM in a sample, or a high IgG titer (HI ≥ 1,280), or a positive NS1 antigen test (ELISA or rapid test).

Patients with incomplete records, insufficient data, or suspected but unconfirmed cases of dengue were excluded from the study. Patients seen on an outpatient basis or with pre-existing neurological disorders were excluded. Additionally, patients with known HIV infection or a positive thick smear for *Plasmodium falciparum* were excluded. Data on sociodemographics, extra-neurological and neurological clinical manifestations, biological test results, and brain computed tomography (CT) scan results were collected for each patient, as well as data on clinical progression during hospitalization.

The data were collected from the patients’ clinical records, as well as from the hospitalization and discharge registers and death records. The data were entered and analyzed using Stata 16 software. The results were expressed as percentages and/ or averages. To determine factors independently associated with in-hospital deaths, we performed univariate and multivariate analyses using logistic regression. In-hospital death occurrence or non-occurrence was the dependent variable, and sociodemographic, clinical, biological, and etiological characteristics were the independent variables. Variables with p ≤ 0.20 in the univariate analysis were included in the multivariate analysis. The significance threshold was p < 0.05. The study was approved by the institutional ethics committees of Tengandogo, Bogodogo and Yalgado Ouédraogo university hospitals and authorized by their administrations (authorizations no. 22024_037_/MSHP /SG/ CHU-T /DG dated 20/02/2024; no. 2024_0295_/MSHP /SG/CHUYO /DG/ DSP du 29/02/2024; no. 2024 / 000028 / MSHP /SG/CHU-B/DG dated 20/02/2024). The confidentiality of the collected data and the anonymity of the patients were guaranteed.

Severe dengue was defined by the presence of one or more of the severity criteria established by the WHO in its 2009 classification:

1. Plasma leakage: Hemoconcentration (hematocrit greater than 45%), shock, and/or serous effusion.
2. Severe hemorrhage: Diffuse and/or profuse hemorrhage with blood loss requiring a blood transfusion.
3. Organ complications: Transaminases (ALT or AST) greater than 1,000 U/L, central nervous system (CNS) involvement, severe thrombocytopenia less than 20,000 cells/mm³, and/or acute renal failure (serum creatinine greater than 176.8 micromoles/L according to the RIFLE classification) [35].

For patients diagnosed with confirmed dengue, the following definitions of neurological complications were applied [9,14,33]:

- Dengue encephalopathy. Signs of brain damage associated with at least one complication related to dengue, such as liver failure, metabolic acidosis, acute renal failure, severe hyponatremia (sodium level less than 120 mmol/L), prolonged shock, disseminated intravascular coagulation, cerebral hemorrhage, or cerebral edema on a brain CT scan, in the absence of other causes, and with normal CSF (cytology, proteinorachia, and microbiological study).
- Probable DENV encephalitis: Clinical signs of brain involvement associated with CSF pleocytosis and elevated proteinorachia in the absence of other neuroinvasive pathogens (normal results from bacteriology, parasitology, and mycology examinations or cultures).
- Stroke. Sudden onset of focal neurological deficit in cerebral vascular territory, confirmed by cerebral CT scan as ischemic or hemorrhagic, after excluding classic causes of stroke.
- Acute disseminated encephalomyelitis. Suspected when clinical signs of brain and/ or spinal cord involvement appear and confirmed by magnetic resonance imaging (MRI) of the brain and spinal cord.
- Other central nervous system (CNS) disorders: The appearance of signs of CNS involvement is confirmed by a CT scan and/ or CSF analysis.
- Peripheral nervous system damage. The appearance of a peripheral neurogenic syndrome in a patient with confirmed dengue fever is indicative of Guillain-Barré syndrome and can be confirmed by electroneuromyography (ENMG) and CSF albumin-cytological dissociation.
- Muscle involvement: The appearance of myogenic syndrome, confirmed by elevated muscle enzymes and/or myogenic syndrome on ENMG.

## Results

A total of 1,057 patients were hospitalized for confirmed dengue cases between January 1 and December 31, 2023 at our study sites: 76 cases confirmed by positive IgM ELISA serology (7.2%), 309 cases confirmed by virus detection by RT-PCR (29.2%), and 672 highly presumptive cases (increased IgG titer + positive NS1 antigen (ELISA or rapid test) (63.6%). Among these cases, 116 presented with neurological complications, representing a prevalence of 11%. All of these were confirmed cases of dengue fever, either by positive IgM ELISA serology in 23 cases (19.8%) or by RT-PCR in 93 cases (80.2%).

The average age of patients was 47.6 ± 1.94 years (range 16 to 95 years). The 20-50 age group included 49 patients (42.2%), and 60 patients (51.7%) were female. Hypertension (HT) with 33 cases (28.4%), type 2 diabetes with 13 cases (11.2%), and sickle cell disease with 7 cases (6%) were the most common comorbidities (Table I).

**Table I:** Distribution of patients hospitalized for dengue with neurological complications according to sociodemographic characteristics and comorbidities

Characteristic	Headcount (n = 116)	Percentage
Age		
≤ 50 years	62	53,4
50-80 years	47	40,5
≥ 80 years	7	6,0
Gender		
male	60	51,7
female	56	48,3
History Comorbidities		
hypertension	33	28,4
type 2 diabetes	13	11,2
sickle cell disease (SS, SC, CC)	7	6,0
asthma	4	3,4
stroke sequelae	3	2,6
peptic ulce	2	1,7
other	4	3,4

The main reasons for hospitalization were consciousness disorders with 50 cases (43.1%), severe digestive symptoms with 35 cases (30.2%), hemorrhagic symptoms with 26 cases (22.4%), repetitive seizures with 22 cases (19%), and respiratory distress with 21 cases (18.1%) (Table II).

**Table II:** Distribution of patients with dengue-related neurological complications according to the reason for hospitalization

Reason for hospitalization	Headcount (n = 116)	Percentage (%)
Severe persistent infectious syndrome	18	15,5
Hemorrhagic signs	26	22,4
Severe digestive signs	35	30,2
Psychomotor agitation	17	14,6
Consciousness disorders	50	43,1
Alteration of general condition	20	17,2
Repeated seizures	22	19
Transient loss of consciousness	4	3,4
Respiratory distress	21	18,1
Focal neurological signs	11	9,5

The average time between the onset of the disease and admission to the hospital was 12.6 days ± 5.2 (6–21 days).

On clinical examination, infectious signs, pain signs, and digestive signs, with 114 cases (98.3%), 104 cases (89.7%), and 49 cases (42.2%) respectively, were the most frequently found signs of extra-neurological involvement. Disturbances of consciousness, with 98 cases (84.5%), epileptic seizures with 24 cases (20.7%), neurological signs of focalization with 19 cases (16.4%), and psycho-behavioral disorders with 17 cases (14.6%), were the most frequently encountered neurological clinical signs (Table III).

**Table III:** Distribution of hospitalized patients with dengue and neurological complications according to extraneurological manifestations and neurological system involvement (n = 116)

Signs of involvement of other organs and the nervous system	Headcount (n = 116)	Percentage (%)
Signs of infection	114	98,3
Signs of pain	104	89,7
Digestive signs	49	42,2
Hemorrhagic syndrome	26	22,4
Alteration in general condition	24	20,7
Pleural effusion and/or ascites	21	18,1
State of shock	7	6
Neurological signs		
Impaired consciousness	98	84,5
Glasgow ≤ 8	31	26,7
Glasgow [9-14]	67	57,8
epileptic seizures	24	20,7
psycho-behavioral disorders	17	14,6
Neurological signs of focalization	19	16,4
hemiplegia/hemiparesis/tetraparesis	19	16,4
aphasia of expression	2	1,7
frontal syndrome	1	0,9
peripheral neurogenic syndrome	1	0,9
meningeal syndrome	3	2,6
intracranial hypertension syndrome	1	0,9

Biologically, we observed acute hepatic cytolysis syndrome, acute renal failure, and thrombocytopenia in 76 cases (65.5%), 54 cases (46.5%), and 44 cases (37.9%), respectively (Table IV).

**Table IV:** Distribution of hospitalized patients with dengue and neurological complications according to laboratory abnormalities and brain CT findings (n = 116)

Biological abnormalities	Headcount (n = 116)	Percentage (%)
Anemia	40	34,5
Hyperleukocytosis	46	39,6
Leukopenia	6	5,2
Thrombocytopenia	44	37,9
Increased hematocrit	40	34,5
Acute renal failure	54	46,5
Acute hepatic cytolysis	76	65,5
Hyperglycemia	22	19
Increased CR	59	50,9
Hypocalcemia	41	35,3
Hyponatremia	31	26,7
Hypernatremia	2	1,7
Hypokalemia	18	15,5
Hyperkalemia	12	10,3
Hypomagnesemia	9	7,7
Brain CT scan	(n = 23)	
Stroke	7	6
Ischemic	2	1,7
Intracerebral hemorrhage	5	4,3
Encephalitis	1	0,9
Cortico-subcortical atrophy	2	1,7

Brain CT scans were performed on 23 patients (19.8%) exhibiting warning signs, revealing stroke in seven patients (6%).

According to the WHO classification of dengue clinical cases [35], of the 116 patients hospitalized for dengue with neurological complications, 98 (84.5%) had severe dengue, while 18 (15.5%) had dengue with warning signs. The most common finding among clinical cases of severe dengue was impaired consciousness, with 98 cases (84.5%), followed by severe organ damage with 76 cases (65.5%) (Table V).

**Table V:** Distribution of hospitalized patients with dengue and neurological complications according to the 2009 WHO clinical classification of dengue (n = 116)

Classification des cas cliniques de dengue selon l’OMS	Headcount (n = 116)	Percentage (%)
Dengue with warning signs	18	15,5
Severe dengue	98	84,5
alertness disorders	98	84,5
plasma leakage syndrome	40	34,5
with respiratory distress (pleural effusion, ascites, edema)	24	20,7
with shock	7	6
severe hemorrhage requiring blood transfusion	12	10,3
severe organ damage (isolated or associated)	76	65,5
severe hepatic cytolysis (ASAT or ALAT >10,000 U/L)	32	27,6
severe acute renal failure (creatinine > 300 µmol/L)	28	24,1
severe thrombocytopenia	37	31,9

A lumbar puncture with CSF analysis and an ENMG confirmed demyelinating sensorimotor Guillain-Barré syndrome (GBS) in a patient who presented with an ascending peripheral sensorimotor neurogenic syndrome.

The clinical spectrum of neurological complications in patients hospitalized for dengue included metabolic encephalopathy (9.4%), probable DENV encephalitis (0.8%), stroke (0.7%), and GBS (0.1%) (Table VI).

**Table VI:** Clinical spectrum of neurological complications in hospitalized dengue patients (n = 1 057) and in those with neurological complications (n = 116)

Clinical spectrum of neurological complications of dengue	Headcount		Percentage (%)
	**116**	**1 057**	
Metabolic encephalopathy	99	85,3	9,4
Probable DENV encephalitis	9	7,8	0,8
Stroke	7	6,0	0,7
Intracerebral hemorrhage	5	4,3	0,5
ischemic	2	1,7	0,2
Guillain-Barré syndrome	1	0,9	0,1

The emergency department (63 hospitalized patients, 54.3%) and the intensive care unit (26 cases, 22.4%) were the main departments for hospitalization (Table VII).

**Table VII:** Distribution of patients with neurological complications according to the direct admitting department

Service d’hospitalisation / Hospitalization Department	Effectif / Headcount (n = 116)	Pourcentage / Percentage (%)
Service d’accueil des urgences médicales / Emergency Department	63	54,3
Service de réanimation / Intensive Care Unit	26	22,4
Service des maladies infectieuses / Infectious Diseases Department	19	16,4
Service de médecine interne ou de neurologie / Internal Medicine or Neurology Department	8	6,9

Treatment consisted mainly of analgesics for 114 patients (98.3%), antibiotics for 86 patients (74.1%), oxygen therapy for 58 patients (50%), and red blood cell or platelet transfusions for 32 patients (27.6%) (Table VIII).

**Table VIII:** Distribution of patients hospitalized for dengue with neurological complications according to treatments received

Traitements reçus / Treatments received	Effectif / Headcount (n = 116)	Pourcentage / Percentage (%)
Antalgiques / Analgesics	114	98,3
Antibiotiques / Antibiotics	86	74,1
Oxygénothérapie / Oxygen therapy	58	50,0
Transfusion (culots globulaires ou plaquettaires) / Transfusion (red blood cells or platelets)	32	27,6
Hémostatiques / Hemostatics	25	21,5
Antiémétiques / Antiemetics	19	16,4
Anti HTA / Anti-hypertensives	18	15,5
Antiépileptiques / Antiepileptics	17	14,7
Antidiabétiques / Antidiabetics	17	14,7
Antipsychotiques / Antipsychotics	9	7,8
Hémodialyse / Hemodialysis	5	4,3
Antidépresseurs / Antidepressants	5	4,3
Antiagrégants plaquettaires / Antiplatelet agents	5	4,3
Antiœdémateux / Anti-edema drugs	3	2,6
Corticothérapie / Corticosteroid therapy	2	1,7
Immunoglobulines / Immunoglobulins	1	0,9
Héparinothérapie curative / Curative heparin therapy	1	0,9
Antiviraux / Antivirals	1	0,9

The average length of hospital stay was 4.5 days ± 0.5 days. A total of 80 patients died in the hospital, representing a hospital case fatality rate of 69%. Ten patients (9%) were lost to follow-up. The remaining 26 patients survived, 12 of whom had neurological sequelae at discharge.

In the bivariate analysis, the following were significantly associated with in-hospital death: coma (p = 0.02), seizures (p = 0.03), altered mental status (p = 0.00), desaturation (p = 0.00), and acute renal failure (p = 0.00). Using stepwise logistic regression in multivariate analysis, acute renal failure (OR = 3.49; p = 0.02) was the only factor independently associated with death (Table IX).

**Table IX:** Results of univariate and multivariate analyses for the identification of sociodemographic, clinical, and biological variables significantly associated with in-hospital case fatality rate

	Analyse univariée / Univariate analysis			Analyse multivariée / Multivariate analysis		
Variables / Variables	Total / Total	Décès / Deaths	p-value / p-value	OR / OR	95% IC / 95% CI	p-value / p-value
Âge > 50 ans / Age > 50 years	116	80	-			
Sexe masculin / Male	56	34	0,07			
Sexe féminin / Female	60	46	0,07			
Antécédents d’HTA / History of hypertension	33	24	0,58			
Diabète type 2 / Type 2 diabetes	13	10	0,51			
Antécédents d’AVC ischémique / History of ischemic stroke	3	2	0,93			
Déficit moteur / Motor deficit	16	10	0,55			
Signes infectieux / Signs of infection	43	33	0,17			
Poussée d’HTA lors de l’admission / Hypertensive crisis on admission	28	19	0,88			
Troubles de la vigilance (Glasgow ≤ 14) / Alertness disorders (Glasgow ≤ 14)	98	75	0,00			
Coma (Glasgow ≤ 8) / Coma (Glasgow ≤ 8)	31	27	0,02	2,49	0,65 – 9,44	0,18
Désaturation (SpO2<95%) / Desaturation (SpO2<95%)	61	50	0,00	2,37	0,41 – 6,76	0,24
Crises épileptiques / Epileptic seizures	24	12	0,03	0,82	0,17 – 3,86	0,80
Insuffisance rénale aiguë / Acute renal failure	54	47	0,00	3,49	1,19 – 10,27	0,02
Cytolyse hépatique / Hepatic cytolysis	76	55	0,27			
Hyperglycémie / Hyperglycemia	22	18	0,09			
Thrombopénie / Thrombocytopenia	44	32	0,33			
Complication neurologique type encéphalopathie métabolique / Neurological complication such as metabolic encephalopathy	99	76	0,00	1,49	0,28 – 7,80	0,64

## Discussion

Neurological manifestations associated with dengue infections are becoming more prevalent, especially during epidemics. These manifestations pose a challenge to medical practice and have an incidence rate ranging from 0.5% to 20%, according to recent literature [1,9,13,25,33,38-41,48]. The spectrum of dengue-related neurological manifestations is commonly divided into three categories:

a. Complications related to the direct neurotropism of the dengue virus, including encephalitis, meningitis, myelitis, and myositis
b. Systemic complications related to dengue infection, including encephalopathy, stroke, and hypokalemic paralysis
c. Post-infectious immune-mediated complications, primarily acute disseminated encephalomyelitis GBS, and optic neuritis [9,14,24,33,38].

DENV encephalitis is a common neurological complication of dengue infection in various parts of the world [18,24,34,43,44]. In our study, however, DENV encephalitis was found in only 0.8% of hospitalized dengue patients.

The diagnostic criteria for dengue virus (DENV) encephalitis are based on the presence of fever and acute signs of brain involvement. This is associated with the detection of IgM-type anti-DENV antibodies, DENV RNA, or NS1 antigen in serum and/or CSF, after excluding other causes of viral encephalitis and encephalopathy [43]. However, computed tomography (CT) and magnetic resonance imaging (MRI) scans are not always helpful, as they may appear normal. In our study, we considered a diagnosis of DENV encephalitis in patients with signs of brain involvement within 30 days of dengue fever onset, confirmed by the presence of IgM anti-DENV antibodies, DENV RNA, or NS1 antigen in serum. The diagnosis was confirmed by clear CSF with pleocytosis and hyperproteinorrachia, as in the series by Soares *et al.* [43] and Carod-Artal *et al.* [9], after excluding other causes of encephalitis with clear CSF through bacteriological, parasitological, and mycological examinations of the CSF and negative HIV serology. However, markers of DENV infection in the CSF, such as IgM anti-DENV antibodies, DENV RNA by PCR, or NS1 antigen, could not be detected due to the unavailability of these tests in our setting. However, normal CSF cellularity and protein levels are insufficient to rule out DENV encephalitis. Markers of DENV encephalitis in the CSF are essential for a definitive diagnosis, particularly in cases of DENV encephalitis without CSF pleocytosis, which account for 20% of cases [2,7]. The unavailability of methods to detect these markers has likely contributed to their underdiagnosis in our study. Recently, Fong *et al.* proposed more stringent diagnostic criteria: detection of DENV in CSF and/or brain tissue is required for a definitive diagnosis of DENV encephalitis, while detection of anti-DENV IgM in CSF is required for a probable diagnosis [14].

Other neuroinvasive central nervous system (CNS) infections with dengue virus (DENV), such as meningitis and myelitis, are reported less frequently. This is evidenced by our series, in which no such cases were found [39,43,49].

Dengue-associated encephalopathy is the most common neurological manifestation of dengue [9,33,39]. It is diagnosed based on signs of brain dysfunction [9,39]. The diagnosis is confirmed by normal CSF studies (cytology, proteinorachia, and glycorachia) [9]. Dengue-related encephalopathy results from multiple etiological factors, including cerebral edema, cerebral hypoxia and/or hemorrhage, and associated systemic dysfunctions, such as prolonged shock, profound hyponatremia, liver failure, renal failure, metabolic acidosis, disseminated intravascular coagulation, and the release of toxic substances (cytokine storm), which is associated with vascular permeability syndrome [9,16,21,24,33].

The incidence of dengue-related encephalopathy varies depending on the population studied (adults or children) and the study [9,24]. In our study, we recorded encephalopathy in 9.4% of hospitalized dengue patients, compared to 2.5% in Thailand [36], 0.8% in India [24], and 0.5% in Vietnam [8]. The high incidence of dengue-associated encephalopathy in our study could be explained by the recruitment of clinical cases of severe dengue (84.5%) from tertiary referral hospitals in Ouagadougou during an epidemic. Patients were admitted late, often at a stage when complications were intertwined and multiple organ failure occurred. In our study, neurological complications coexisted with severe organ damage (65%), plasma leak syndrome (34.5%), and severe hemorrhage requiring blood transfusion (10.3%). GBS is reported to be the most common immune-mediated, post-infectious complication [27]. In a study by Verma *et al.* in India [47] and Jackson *et al*. in Jamaica [18], GBS accounted for 11.5% and 3.7%, respectively, of patients with dengue-related neurological complications. However, in our study, GBS accounted for only 0.9% of dengue-related neurological complications. The low proportion of GBS cases in our study could be due to the short hospital stay, averaging 4.5 days, whereas post-dengue GBS onset occurs 5–15 days after infection [15,46]. Post-dengue GBS is characterized by a predominance of demyelinating

[9] and axonal sensorimotor forms [9,15], as was found in our study. The mechanism of GBS associated with DENV involves molecular mimicry between human proteins related to GBS and the DENV polyprotein [50,51]. The authors recommend systematic screening for recent DENV infection in cases of GBS in endemic areas [47]. Other post-infectious dengue syndromes with proven or strongly suspected immune mediation that have been reported in the literature were not observed in our study due to a lack of appropriate diagnostic tools, particularly brain MRI and DENV PCR in CSF. These syndromes are mainly acute disseminated encephalomyelitis and optic neuritis [14,20]. Other dengue fever syndromes, the mechanisms of which are unclear, were also absent from our study: cerebellitis [12], acute transverse myelitis [3], and myositis [31].

Cerebrovascular complications, such as intracranial hemorrhages and ischemic strokes, have been reported during dengue infections, though with low incidence rates. The most frequently reported types of intracranial hemorrhages are intraparenchymal, subdural, and subarachnoid hemorrhages [9,24,42]. In our study, 0.5% of patients hospitalized with dengue fever presented with an intraparenchymal hemorrhage. Similar frequencies have been reported in other hospital studies: 0.1% to 0.3% in India [24,30] and 1.8% in Pakistan [32]. Current data suggest that intracranial hemorrhages complicating dengue fever may result from the overproduction of pro-inflammatory cytokines, which are responsible for immune-mediated endothelial cell damage, vasculopathy, thrombocytopenia, and platelet dysfunction. These factors cause a hemorrhagic diathesis [5,24].

There have been rare cases of ischemic stroke complicating dengue fever described in the literature [24,47] and in our series. Possible mechanisms include hemostasis dysfunction, severe volume depletion, hypotension, meningovascularitis, and myocarditis [24].

Including dengue diagnostic tests in the etiological assessment of stroke in dengue-endemic areas could lead to a better understanding of its cerebrovascular complications.

In Asian and South American countries, mortality due to dengue infection ranges from 3% to 5% [39], whereas mortality in patients who develop a dengue-associated neurological complication varies from 5% to 30% [17,28,32,40]. In Burkina Faso, an in-hospital case fatality rate of 7.8% was reported among patients hospitalized for severe dengue in 2016; neurological complications accounted for 63.4% of deaths [45]. Similarly, another retrospective study conducted in Brazil found that 48.8% of dengue-related deaths were due to neurological complications [2]. A study conducted in Pakistan found that the occurrence of neurological complications in patients hospitalized for dengue was a significant predictor of death [32].

Our study confirms the high case fatality rate attributable to neurological complications of dengue, particularly in our context, with fatality rates for dengue and cases of dengue with neurological complications of 10% and 69%, respectively. Several factors could explain this unfortunate situation. These include late admission and treatment of patients with sometimes life-threatening complications (e.g., impaired consciousness, coma, status epilepticus, respiratory distress, massive hemorrhage, disseminated intravascular coagulation, etc.). These complications require specialized emergency care. The average time between symptom onset and hospitalization for our patients was 12 days, whereas dengue fever complications appear around the seventh day after symptom onset.

The causes of these delays in the admission and treatment of complicated dengue cases include:

a. high frequency of self-medication among the population, with patients only seeking medical attention when complications become severe [50];
b. insufficient systematic use of biological diagnostic tests for malaria (thick and thin smears) and dengue (rapid detection tests for the dengue NS1 antigen, as well as for IgG and IgM antibodies). In a context of dual endemicity of malaria and dengue in Burkina Faso, this shortcoming contributes to confusion or diagnostic uncertainty, leading to delays in diagnosis and adequate treatment [6]. Additionally, dengue diagnostic tests were unavailable in 2023 due to a global shortage, particularly in Africa and the Sahel region, which corresponds with the period of the dengue epidemic in Burkina Faso [29];
c. insufficient availability of intensive care beds in Ouagadougou’s university hospitals, combined with their inaccessibility due to financial difficulties families face when covering hospitalization and care costs in the absence of universal health insurance in Burkina Faso, a low-income country [19]. Consequently, only 22% of our patients were transferred to intensive care, compared to 80% who were eligible;
d. finally, there are difficulties in providing medical care for complicated forms of the disease due to their complexity, the unavailability of certain therapeutic resources, and the absence or lack of knowledge of protocols for caring for complicated dengue.

To reverse this trend, we recommend the following to the Ministry of Health authorities of in Burkina Faso:

a. continue and intensify primary dengue prevention through vector control;
b. accelerate the accessibility and availability of rapid diagnostic tests for dengue and malaria;
c. produce and disseminate clear regulatory guidelines and protocols for managing dengue cases at the community level and at various levels of the national health system;
d. include dengue treatment resources among essential medicines and consumables.

In our study, acute renal failure was identified as an independent risk factor for in-hospital mortality in patients hospitalized for dengue with neurological complications (OR = 3.49; p = 0.02). A similar finding was made in other studies [26,28]. Acute renal failure (ARF) is a frequent and severe complication of dengue associated with a high case fatality rate [11,34,45]. ARF contributes significantly to the increased incidence and case fatality rate of dengue’s neurological complications, particularly through the uremic encephalopathy it causes [11,28].

## Conclusion

Our study of the 2023 dengue epidemic in Burkina Faso revealed that over 10% of hospitalized adult patients experienced neurological complications, primarily metabolic encephalopathy, followed by probable DENV encephalitis, and rare instances of stroke and GBS. The clinical course was burdened by excess mortality in approximately seven out of 10 cases, exacerbated by the coexistence of ARF. To reverse this trend, early and appropriate hospital care must be organized in intensive care units equipped to treat severe dengue cases. Additionally, operational research must be conducted to identify, prevent, and manage the predictors of dengue complications, particularly neurological ones. This must be done without neglecting prevention through intensified vector control, improved availability and accessibility of rapid dengue diagnostic tests, and application of national treatment guidelines and protocols at all levels of the healthcare system.

## Funding

The study did not receive funding.

## Authors’ contributions

LOMPO Djingri Labodi: study design, documentary research, data collection, development of the study protocol, data analysis, and manuscript writing.

KAGAMBEGA, D. Sylvie: Data collection, data analysis, documentary research, and manuscript writing.

OUATTARA Souleymane: Data collection, protocol review and validation, manuscript review. GNAMPA Melody Zeinab: Data collection.

KERE M. Fabienne Y.: Data collection.

NAPON Christian: validation of the study protocol and supervision of the study.

MILLOGO Athanase: Protocol validation and supervision.

## Conflicts of interest

The authors declare no conflicts of interest
